# Investigation on Aluminum Alloy Reflector Mirror Processing Technology Combining Ultrasonic Rolling and Single-Point Diamond Turning

**DOI:** 10.3390/mi15121527

**Published:** 2024-12-22

**Authors:** Yuanhao Ma, Zhanjie Li, Gang Jin, Xiangyu Zhang, Longsi Li, Huaixin Lin, Guangyu Wang, Zhenyu Long

**Affiliations:** School of Mechanical Engineering, Tianjin University of Technology and Education, Tianjin 300222, China

**Keywords:** ultrasonic rolling, single-point diamond turning, grain, reflectivity, surface roughness

## Abstract

In the process of aluminum alloy reflector mirror processing, the structural defects of aluminum alloys present bottlenecks restricting the development of aluminum alloy reflector mirror processing technologies. Therefore, this study proposes an aluminum alloy reflector mirror processing method involving ultrasonic rolling and single-point diamond turning. The core idea of this method is to use ultrasonic rolling to pretreat the surface of the workpiece to refine the grains and increase the hardness, then perform single-point diamond turning to improve the optical reflection performance. In this study, an ultrasonic rolling cutting experiment was carried out, and the influence of the material preparation method on the microstructure and hardness of the workpiece was analyzed. An ultrasonic rolling single-point diamond turning experiment was carried out, and the influence of the material preparation method on the reflection performance of single-point diamond turning was studied. Results showed that compared with single-point diamond turning after ordinary milling, the ultrasonic rolling single-point diamond turning method has certain advantages in improving the surface reflection performance, with an increase of 5.116%. The method proposed in this study can provide new ideas for the high-quality processing of aluminum alloy reflector mirrors.

## 1. Introduction

With the continuous development of aerospace systems and astronomical observation systems, metal materials (such as aluminum alloys, titanium alloys, and magnesium alloys) with good comprehensive properties and low material cost have been widely used [1,2]. Among them, aluminum alloys have been widely used in aerospace optical systems due to their low cost, easy processing, high strength, and corrosion resistance [3,4].

In recent years, with the increasing requirements for machining accuracy, surface modification technology has been widely developed. On the basis of the principle of severe plastic deformation (SPD), researchers have developed a variety of surface modification techniques, such as shot peening [5,6], ultrasonic shot peening [7,8], laser shot peening [9,10], surface mechanical friction treatment (SMAT) [11,12], rolling [13,14], and ultrasonic rolling [15,16]. After surface modification treatment, the surface microstructure of the material changes, such as the grain refinement, which improves the mechanical properties and corrosion resistance [17,18,19,20,21]. Among them, ultrasonic surface rolling processing (USRP) is a dynamic rolling technology that combines ultrasonic impact and traditional rolling [22]. Compared with RP and other surface modification technologies, USRP can not only achieve surface modification and improve yield strength but also process the surface texture and reduce tool wear, as an appropriate texture can improve the friction and wear performance of the workpiece [23,24]. Fan et al. [25] combined USRP and laser-assisted processing in carrying out cutting experiments on TC11 titanium alloy. The results showed that the USRP process greatly reduces scratches and processing defects, reduces grain size, and improves surface processing quality. Therefore, USRP can effectively reduce the surface roughness, refine grains, and improve surface machining accuracy, and its grain refinement is the main factor to improve surface hardness and corrosion resistance [26,27].

Single-point diamond turning (SPDT) is an ultra-precision machining technology which can achieve submicron or even nanoscale machining accuracy [28]. It is often used to process materials such as metals and polymers. With the development of SPDT technology, it has been able to process ultra-smooth surfaces with surface accuracy less than 1 nm and is widely used in the field of optical component processing [29]. Tan et al. [30], based on additive manufacturing technology, used the single point diamond turning process to process aluminum reflector mirrors. The study showed that the reflector mirror surface roughness *Ra* reached 8 nm after gold film was coated on the surface, which met the requirements of the space environment and showed good optical properties in the infrared band. Ran et al. [31] predicted the appropriate feed speed and cutting speed of single-point diamond turning by establishing a mixed damage model and used the pre-strain method of a split Hopkinson pressure bar (SHPB) to calculate the unknown coefficient of the model, revealing and predicting the fracture caused by the size effect in SPDT. Zhou et al. [32] analyzed the surface morphology and cutting force of single-point diamond cutting in a micro-lubrication environment (MQLM-oil), a dry environment (ODM), and a high-pressure gas environment (HGM), and concluded that better surface quality could be obtained in the micro-lubrication environment (MQLM-oil). Chen et al. [33] analyzed and studied the mechanism of straight-end cutting tools in the processing of Zerodur optical components and found that compared with traditional round-end cutting tools, the surface roughness could be reduced from 173 nm to 15 nm by using straight-end cutting tools. Aiming at the influence of machine tool vibration on mirror roughness, Zhuang et al. [34] developed a dynamic model of the Z-direction vibration of ultra-precision machine tools and verified the correctness of the model through experiments. It was found that this method could reduce the vibration intensity by about 50%, and this method was applied to the actual processing of aluminum alloy mirrors. To improve the surface processing quality of a microlens array, Du et al. [35] proposed a laser-assisted slow tool servo diamond turning process (LA-STSDT) by combining laser assistance with SPDT. The experimental results showed that the surface roughness of the LA-STSDT process was reduced by 45.3% compared with traditional slow tool servo diamond turning. To change the machinability of titanium alloys, Yeo [36] proposed a processing method combining magnetic field effects with SPDT. Through experimental research, it was found that this method not only realized the processing of metal mirrors but also reduced surface processing marks and improved surface integrity.

Therefore, based on the above research, it can be said that SPDT is widely applied in the field of mirror machining. However, when it comes to the processing of aluminum alloy mirrors, issues such as deformation and built-up edges due to the softness of the material have been found to affect machining accuracy and the reflectivity of the mirrors. Consequently, inspired by the fact that USRP can achieve surface modification, this study proposes a new composite processing technology combining USRP and SPDT: the workpiece is pretreated by USRP to improve the microstructure of the surface layer of the workpiece, and then SPDT processing is carried out to improve the surface processing quality and further improve the surface optical reflection performance. Through an ultrasonic rolling single-point diamond turning experiment on the 6061 aluminum alloy, the influence of the material preparation method on the microstructure, surface roughness, surface morphology, and optical reflection performance is explored.

## 2. Processing Method

At present, there are four methods for making metal reflector mirrors: single point diamond turning, optical cold processing, additive manufacturing, and replication [37,38,39,40]. Because single-point diamond turning has good controllability and can obtain submicron shape accuracy and nanometer surface roughness, it has gradually become an important method for metal reflector mirrors. The difficulty of aluminum alloy reflector mirror processing is that aluminum alloys have soft textures and low hardness. It is easy to produce mechanical damage during processing and forming, resulting in surface defects such as scratches and wear, which makes the surface flatness and finish inadequate after processing. These problems greatly limit the manufacturing accuracy and application of aluminum alloy reflector mirrors.

**Figure 1:**
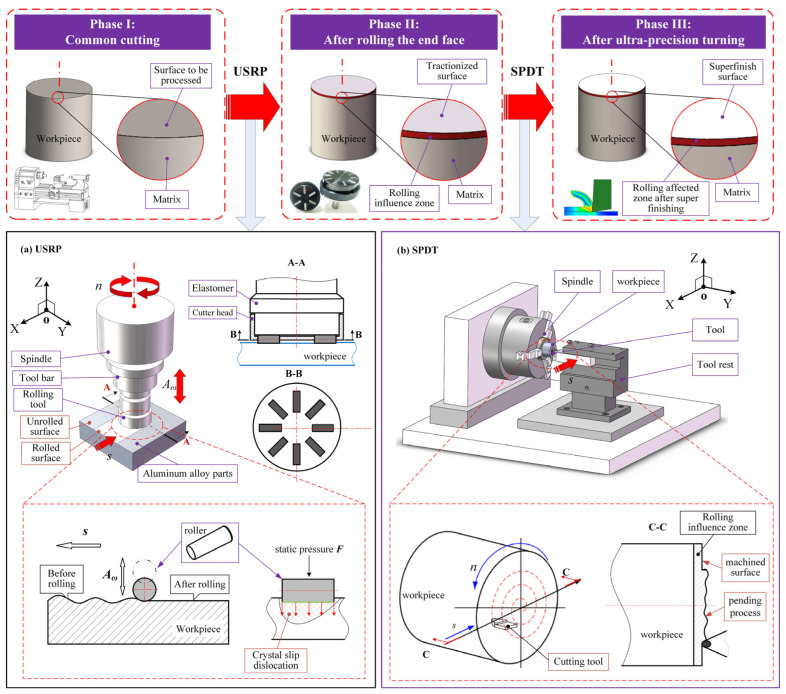
USRP-SPDT schematic diagram. (**a**) Machining process and principle of USRP. (**b**) Machining process and principle of SPDT.

It is known that laser-assisted processing, rolling, ultrasonic rolling, and other technologies can achieve material surface modification and improve machinability. Combined with the practical problems of aluminum alloy reflector mirror processing, this study proposes an ultrasonic rolling single-point diamond turning (USRP-SPDT) composite processing method. The specific processing principle is shown in Figure 1.

(1) USRP: Ultrasonic surface rolling processing (USRP) is a material surface modification method which combines the principles of ultrasonic and conventional rolling processing (RP) to achieve high-precision and high-efficiency machining of workpieces [41]. As shown in Figure 1a, USRP technology includes a spindle cutting system, an ultrasonic vibration cutting system, and a rolling processing system. In the machining process, the rotation of the roller and the feed of the spindle work together to roll the surface of the workpiece, so that the plastic deformation occurs on the surface of the workpiece. Combined with the high-frequency vibration of the ultrasonic system (the vibration direction is *A_ω_*), the surface of the workpiece is impacted, resulting in crystal slip dislocation and grain refinement, so that the surface hardness of the workpiece is improved. Studies have shown that combining USRP with laser additive manufacturing processes can improve the forming quality of metal additive manufacturing [42]. Therefore, this study uses USRP technology to create a refined grain layer on the surface of aluminum alloys, achieving a new microstructure on the surface layer and hardening the machined surface, thereby enhancing the machinability of aluminum alloys and subsequently improving the processing accuracy of SPDT.

(2) USRP-SPDT: Single-point diamond turning (SPDT) is an ultra-precision machining technology. It can achieve high-precision machining of the workpiece only by turning and can improve the machining efficiency [43]. As shown in Figure 1b, a grain refinement layer is generated on the surface of the workpiece by USRP technology to improve the hardness of the material, so that the physical properties of the surface microstructure of the material are better than the initial surface structure of the material. SPDT processing is performed in the grain refinement layer area, which may reduce the deformation and built-up edges caused by the softness of the material, thereby obtaining higher processing accuracy and improving the surface optical reflection performance. Theoretical analysis has indicated certain feasibility, which is of great practical significance and research value for improving the surface optical properties of metal reflector mirrors and manufacturing high-level metal reflector mirrors.

## 3. Single-Point Diamond Turning Material Preparation

### 3.1. Experimental Materials and Programs

Aluminum alloys have been widely used in aerospace optical systems due to their low cost, easy processing, high specific strength, and good corrosion resistance [44]. Therefore, this study selected the 6061 aluminum alloy for aerospace as the research object to verify the advantages of the USRP-SPDT method. The workpiece used in the experiment is an aluminum alloy cylindrical part with a diameter of 40 mm and a height of 40 mm. The material composition and properties are shown in Table 1.

To explore the influence of material preparation methods on the surface properties of the material, the rolling process and common milling (CM) process were introduced as the control group. The surface of the workpiece was plane-rolled by a vertical machining center, in which the ultrasonic system adopts the ultrasonic generator of UBT40-33B and the ultrasonic tool holder of BT40-FR32 produced by Suzhou Sunshines Ultrasonic Equipment Co., Ltd., Suzhou, China. The workpiece was milled before rolling to ensure that the initial state of the workpiece surface was the same during rolling. The material preparation scheme is shown in Table 2.

**Figure 2:**
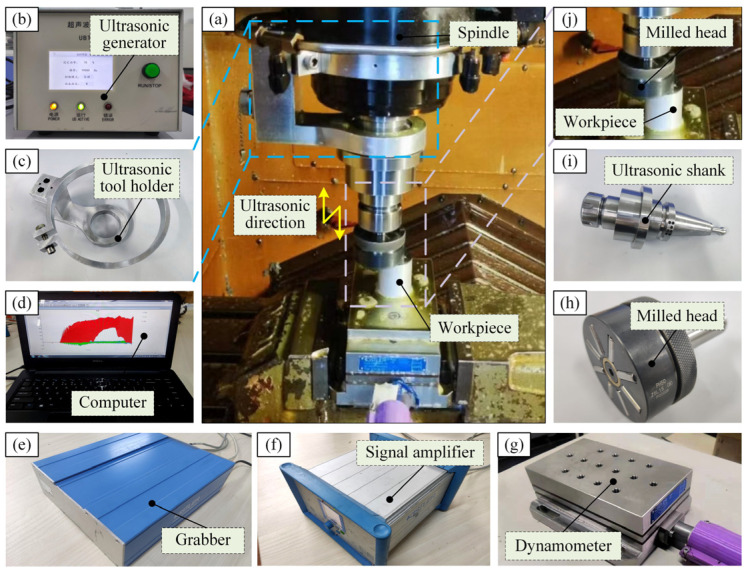
USRP test site. (**a**) is the experimental site, (**b**–**j**) are the components and instruments used in the experiment.

The USRP experiment site is shown in Figure 2. The USRP machining system consists of a spindle machining system, an ultrasonic vibration assisted cutting system, and a force measurement system. The processing platform of the experiment is the Muye S56 vertical machining center produced by China Kunshan Muye Machine Tool Co., Ltd., Kunshan, China. Its stroke is *X* × *Y* × *Z* = 900 mm × 500 mm × 450 mm. The maximum spindle speed is up to 12,000 r/min, and the maximum loading weight is 500 kg. The ultrasonic vibration assisted machining system consists of four parts: the ultrasonic emitter (Figure 2b), the ultrasonic shank (Figure 2i), the rolling head (Figure 2h), and the ultrasonic tool holder (Figure 2c). Among them, the model of the ultrasonic generator is UBT40-33B, the model of the ultrasonic tool holder is BT40-FR32, the ultrasonic amplitude range is 0–4 μm, and the ultrasonic frequency range is 16–40 kHz. The frequency used in this experiment was 19 kHz, and the ultrasonic amplitude was 4 μm. The force measurement system adopted the force measurement system produced by the Swiss KISTLER company, Winterthur, Switzerland. This system consists of four parts: a dynamometer, a charge amplifier, a data acquisition system, and a computer that was used for real-time monitoring of the rolling pressure in this USRP experiment. The force measuring platform is shown in Figure 2g, the model being 9257B and the range 5~10 kN. The charge amplifier is shown in Figure 2f, the model being 5070; its function is to amplify the data obtained by the force platform signal through multiple channels. The data collector is shown in Figure 2e, the model being 5697A; its function is to collect the data amplified by the charge amplifier and compile it into an identifiable digital signal. The computer is shown in Figure 2d; its function is to adjust the time and frequency of the collected signal, display the force data in real-time, and save and process the data.

When preparing the material, the workpiece was installed on the force measuring platform, the ultrasonic tool holder was installed on the spindle of the machine tool, and the ultrasonic shank was fixed on the ultrasonic tool holder. Finally, the rolling head was placed on the ultrasonic shank. By energizing the ultrasonic generator, the generated signal was transmitted to the ultrasonic tool holder to generate a longitudinal vibration trajectory at the rolling head, and the material preparation experiment of SPDT processing began.

### 3.2. Materials Testing

To observe the influence of the material preparation process on the microstructure of the workpiece, metallographic experiments were carried out on the prepared workpiece. In the process of metallographic sample preparation, the sample was first mechanically polished, and then the Kjeldahl solution (1 mL HF + 1.5 mL HCl + 2.5 mL HNO_3_ + 95 mL H_2_O) was used to pre-treat the observation surface for 30 s of corrosion. After corrosion, the metallographic structure of the sample was observed using an MEF3 multifunctional metallographic microscope produced by Leica company of Austria, Germany.

**Figure 3:**
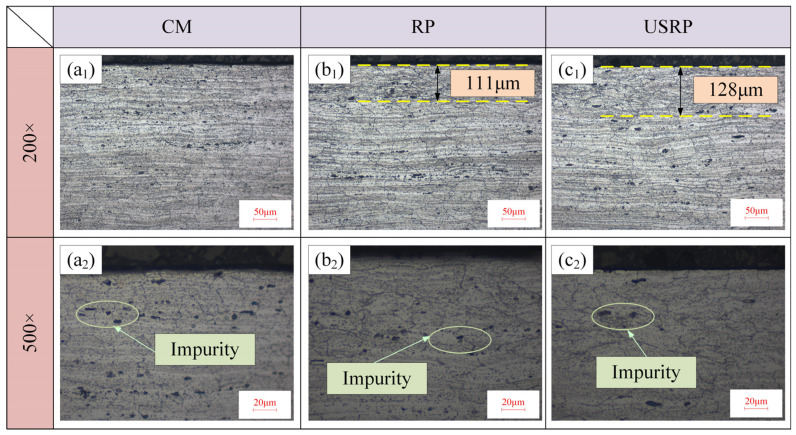
Effect of pretreatment on microstructure. (**a_1_**,**a_2_**) are microstructure observation results at 200× and 500× magnification, respectively, obtained using the CM processing method. (**b_1_**,**b_2_**) are microstructure observation results at 200× and 500× magnification, respectively, obtained using the RP processing method. (**c_1_**,**c_2_**) are microstructure observation results at 200× and 500× magnification, respectively, obtained using the USRP processing method.

**Figure 4:**
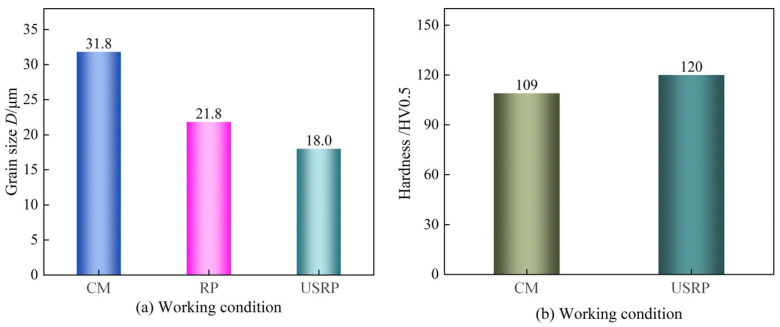
Effect of pretreatment on (**a**) grain size and (**b**) hardness.

Figure 3 shows the effect of material preparation on the microstructure (shooting results at 200 times and 500 times), while Figure 4 shows the effect of material preparation on the grain size and the hardness. As shown in Figure 3(a_1_,a_2_), the grain distribution in the cross-section area of the workpiece processed by CM was uniform, there was no grain refinement layer composed of grain slip and dislocation, and there were more black spots (called “impurities”) in this area. As shown in Figure 3(b_1_,b_2_), there was an obvious grain refinement layer in the cross-sectional area of the workpiece processed by RP, and the depth of the grain refinement layer was about 111 μm, while the impurities were also reduced. As shown in Figure 3(c_1_,c_2_), the depth of the grain refinement layer in the cross-section area of the workpiece processed by USRP was the deepest (up to 128 μm). Compared with RP, the increase in the depth of the grain refinement layer was 15.3%, and the impurities in this area were also the least. As shown in Figure 4, with the precision and efficiency of the processing technology, the average grain size also showed a decreasing trend, and the hardness showed an increasing trend. When CM was used, the average grain size reached the maximum (*D* = 31.8 μm), and the hardness was 109 HV0.5. When USRP was used, the average grain size reached the minimum (*D* = 18 μm), and the hardness also improved (120 HV0.5). Compared with CM, the average grain size of USRP decreased by 43.40%, and the hardness increased by 10.09%. Compared with RP, the average grain size of USRP decreased by 17.4%. Compared with CM, the average grain size of RP decreased by 31.4%. The reason for these results is that RP produces greater pressure on the surface of the workpiece, so that plastic deformation of the surface material of the workpiece occurs, and then the grain dislocation movement intensifies, causing dislocation tangles, accumulation, and other phenomena, forming dislocation walls and dislocation cells, and continuous plastic deformation makes dislocation cells further transform into subgrains. Subgrains form low-angle or high-angle grain boundaries, the degree of grain breakage increases, and the surface grains are refined. In the process of USRP technology, the tool squeezes the surface of the material while high-frequency vibration occurs. The high-frequency effect of the ultrasound makes the grain dislocation more frequent; the degree of grain breakage further increases, so that the grain is further refined, the grain orientation tends to be randomly distributed, and the hardness of the material is improved.

In summary, the grain refinement caused by USRP had a significant effect on the surface hardness and other properties of the material, improved the structural defects of the aluminum alloy 6061 material itself, and obtained better surface quality after rolling. The USRP process was superior to the RP process in terms of the surface grain refinement of the material; that is, USRP can achieve a better grain refinement effect and deeper grain refinement area than RP. At this time, SPDT processing was carried out. The adhesion force and elastic recovery of the surface structure during cutting separation became smaller, and the stick knife phenomenon and built-up edges were alleviated. Therefore, it was easier to obtain higher surface quality with USRP, which had a positive effect on the subsequent SPDT processing.

## 4. Single-Point Diamond Turning Experiment

### 4.1. Experimental Tool

In SPDT machining, the cutting performance of the tool is one of the important factors affecting the cutting process. Reasonable selection of tool materials is an effective way to improve machining accuracy and efficiency. Therefore, this study selected natural diamond (ND) inserts as cutting tools. ND tools are made of the hardest material among known minerals. After fine grinding, the edge profile of a natural single-crystal diamond is less than 50 nm. It has the advantages of high hardness, high sharpness, and strong wear resistance. It is suitable for precision and ultra-precision machining [45], which greatly improves the machining accuracy and machining efficiency in cutting. The tool used in the experiment is produced by Shenzhen Yuhe Optical Precision Tool Co., Ltd., Shenzhen, China, and its number is D10211106. The geometric parameters of the tool are shown in Table 3.

### 4.2. Experimental Scheme

To improve the machining accuracy of aluminum alloy metal reflector mirrors, this study carried out USRP-SPDT machining experiments. It is worth noting that there is an interaction between the experimental parameters of SPDT and the experimental parameters of USRP. Therefore, this study is based on the materials prepared in Section 3. The USRP-SPDT process method is a preliminary exploration. The experimental scheme involved a single-factor experimental analysis method. Table 4 refers to the material preparation experiment: the effects of the three SPDT workpiece preparation methods (CM, RP, and USRP) on surface roughness, surface morphology, and optical reflection performance were compared. Table 5 refers to the USRP-SPDT experiment, which explored the influence of cutting parameters on the surface roughness and optical reflection performance under the USRP-SPDT processing method.

### 4.3. Experimental Conditions and Detection

**Figure 5:**
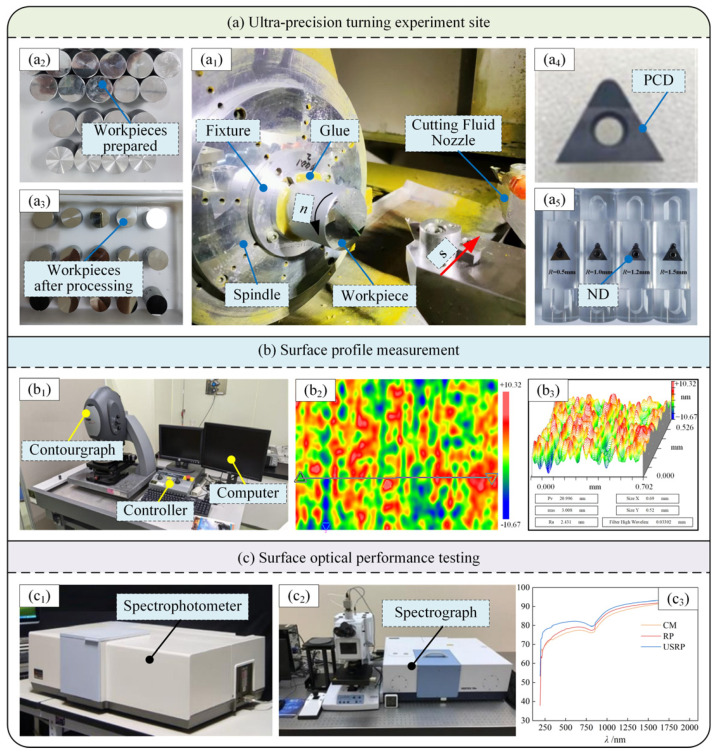
Experimental conditions. PCD refers to the artificial polycrystalline diamond tool, ND refers to the natural diamond tool. (**a_1_**) is the test site, (**a_2_**) is the prepared material, (**a_3_**) is the material after experimental processing, (**a_4_**,**a_5_**) are the tools used in the experiment. (**b_1_**) is a surface profiler, (**b_2_**,**b_3_**) are examples of test results. (**c_1_**,**c_2_**) are surface optical reflectivity detection instruments, and (**c_3_**) is an example of reflectivity detection results.

The experimental conditions of this SPDT experiment are shown in Figure 5. The machine tool used in the experiment was an IL300 ultra-precision CNC high-dynamic diamond lathe. Its stroke is *X* × *Z* = 300 mm × 300 mm, the coordinate axis resolution is 0.03 nm, and the maximum speed is up to 5000 rpm. During the experiment, the fixture (Figure 5(a_1_)) was first fixed on the lathe spindle, and the workpiece was clamped to the fixture. The workpiece was prepared as per Section 3 (Figure 5(a_1_)). Secondly, to reduce the error caused by the circular runout in the turning process, a special glue was used to further secure the connection between the fixture and the workpiece. Finally, the ND tool was installed on the tool holder for the SPDT experiment. Because the surface of the prepared workpiece was hardened and the turning distance was long, to reduce the influence of tool wear on the surface processing quality, the PCD tool (as shown in Figure 5(a_4_)) was used for semi-finishing in the experiment, and then the ND tool (Figure 5(a_5_)) was used for the SPDT experiment. The workpiece after turning is shown in Figure 5(a_3_).

To further explore the turning performance advantages of the USRP-SPDT method, the surface morphology, surface roughness, and optical reflection performance of the workpiece were comprehensively evaluated based on the ultra-precision turning experimental results of the workpiece prepared by CM and RP methods. To facilitate the comparison of the processing quality under different material preparation methods, the different material preparation methods under the same cutting parameters were classified into one group and divided into four groups *A* to *D*. The corresponding processing parameters of groups *A* to *D* were *A*: *n* = 1000 rpm, *s* = 15 mm/min; *B*: *n* = 2000 rpm, *s* = 15 mm/min; *C*: *n* = 3000 rpm, *s* = 15 mm/min; *D*: *n* = 2000 rpm, *s* = 20 mm/min. To reduce the error caused by different observation points of the workpiece, the observation part was uniformly selected as the area at the radius of the workpiece R = 10 mm.

The observation of the surface topography and surface roughness is shown in Figure 5b. The NewViewTM6300 surface profiler (Figure 5(b_1_)) was used, and examples of the observation results are shown in Figure 5(b_2_,b_3_). The detection of the optical reflection performance is shown in Figure 5c; the Lambda900 spectrophotometer (Figure 5(c_1_)) and the VERTEX 70v Fourier transform infrared spectrometer (Figure 5(c_2_)) were used. The measured band range was 190~2000 nm, and an example of the measurement results is shown in Figure 5(c_3_).

## 5. Experimental Results and Discussion

### 5.1. Effect of Material Preparation Method on Surface Quality Achieved by SPDT

(1) Surface morphology and roughness

**Figure 6:**
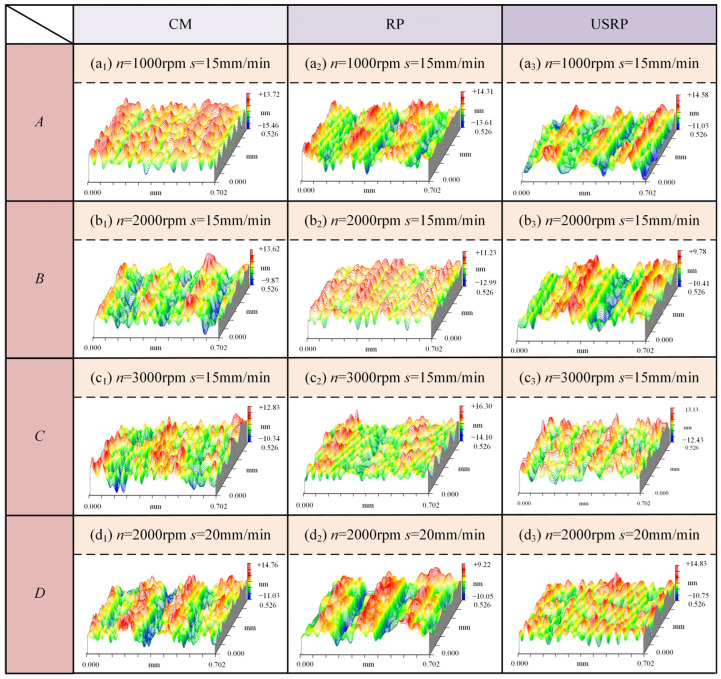
Effect of material preparation method on surface morphology of SPDT. Parameter combinations are as follows: *A*: *n* = 1000 rpm, *s* = 15 mm/min; *B*: *n* = 2000 rpm, *s* = 15 mm/min; *C*: *n* = 3000 rpm, *s* = 15 mm/min; *D*: *n* = 2000 rpm, *s* = 20 mm/min. (**a_1_**–**a_3_**) are the three-dimensional surface topography of CM, RP and USRP under group *A* parameters. (**b_1_**–**b_3_**) are the three-dimensional surface topography of CM, RP and USRP under group *B* parameters. (**c_1_**–**c_3_**) are the three-dimensional surface topography of CM, RP and USRP under group *C* parameters. (**d_1_**–**d_3_**) are the three-dimensional surface topography of CM, RP and USRP under group *D* parameters.

**Figure 7:**
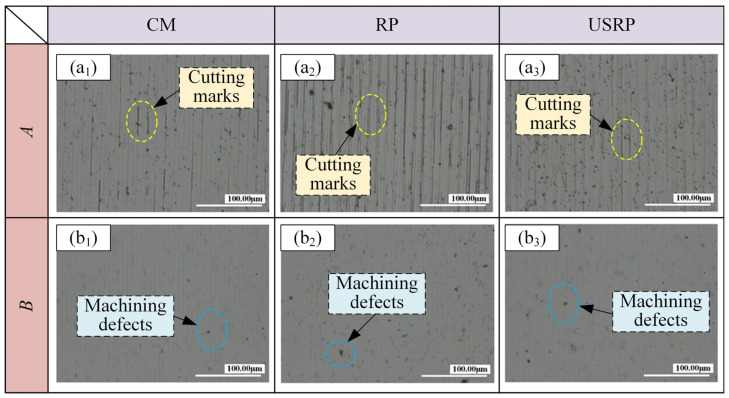
Two-dimensional surface morphology results diagram. Parameter combinations are as follows: *A*: *n* = 1000 rpm, *s* = 15 mm/min; *B*: *n* = 2000 rpm, *s* = 15 mm/min. (**a_1_**–**a_3_**) are the two-dimensional surface topography diagram of CM, RP and USRP under group *A* parameters. (**b_1_**–**b_3_**) are the two-dimensional surface topography diagram of CM, RP and USRP under group *B* parameters.

**Figure 8:**
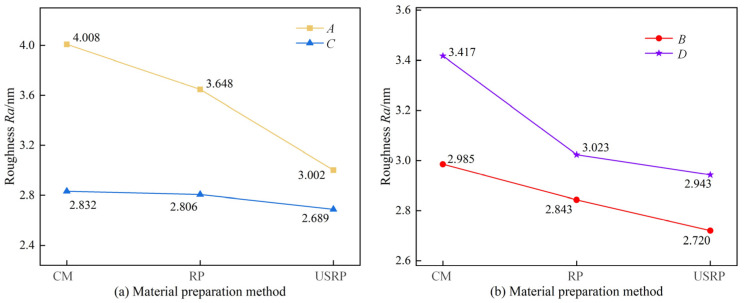
Effect of material preparation method on surface roughness of SPDT. (**a**) shows the measurement results at different speeds, (**b**) shows the measurement results at different feed speeds.

Figure 6 shows the effect of the material preparation method on the surface morphology. Figure 7 shows the results of two-dimensional surface topography. Figure 8 shows the surface roughness measurement results. As shown in Figure 6, under the same parameters, the surface morphology of the workpiece was the worst when machined by CM-SPDT, with uneven peak and valley distribution and the largest undulation, especially in group *A*, where the undulation reached 29.18 nm. The surface morphology of the workpiece was the most gentle when machined by USRP-SPDT, with a uniform distribution of peaks and valleys and the smallest undulation; the undulation was only 25.61 nm under the *A* group of machining parameters, and smaller still for the *B* group, which had the smallest undulation of 20.19 nm. From Figure 7, when USRP-SPDT was used for processing, there were more surface cutting traces, and they were evenly distributed. However, combined with Figure 6, the fluctuation of the cutting traces was smaller, so the surface processing quality was higher. When CM-SPDT was used for machining, the distributio.n of cutting traces was uneven. Combined with Figure 6, the fluctuation of cutting traces was large, so the surface processing quality was low. As shown in Figure 8a,b, the surface roughness of USRP-SPDT was smaller than that of CM-SPDT; under the group A processing parameters, the surface roughness of CM-SPDT was the largest (*Ra* = 4.008 nm), and the surface roughness of USRP-SPDT was the smallest (*Ra* = 3.002 nm). Compared with the former, the surface roughness of the latter was reduced by 25.10%. The reason for these results is as follows: combining Figure 3 and Figure 4 reveals that the microstructure of the workpiece prepared by USRP had changed, the grain size had been greatly reduced and refined, the grain orientation tended to be random, and the hardness of the workpiece had increased, thereby alleviating the problems of build-up edges and machining deformation in the cutting process, thereby reducing the surface roughness achieved by SPDT.

(2) Surface optical reflection properties

**Figure 9:**
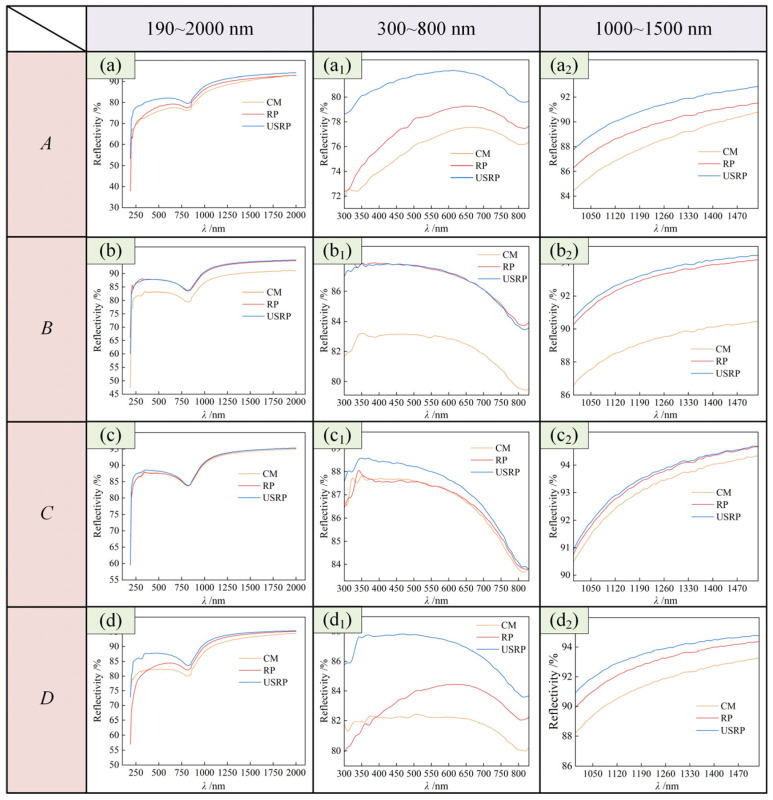
Measurement results for surface optical reflection performance. (**a**–**d**) is the measurement results in the wavelength range of 190–2000 nm. (**a_1_**–**d_1_**) are the measurement results in the short-wavelength range, and (**a_2_**–**d_2_**) are the measurement results in the long-wavelength range.

**Figure 10:**
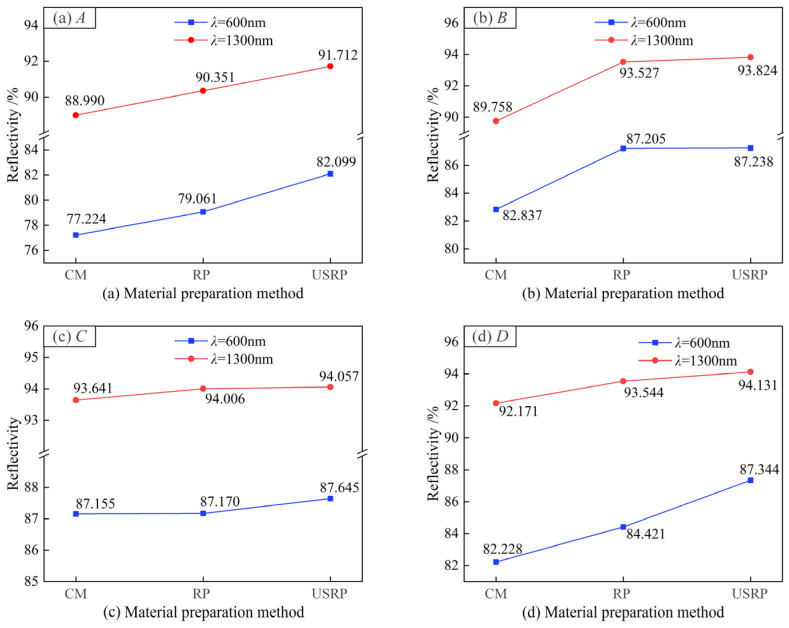
Effect of material preparation method on surface optical reflection properties. (**a**–**d**) shows the influence of the cutting method on the reflectivity of the specific wavelength under the four different parameter combinations of *A*–*D*.

Figure 9 shows the measurement results of surface optical reflection properties. Figure 10 shows the influence of the material preparation method on the surface optical reflection performance. Reflectivity values of *λ* = 600 nm and *λ* = 1300 nm were selected as the reference, and the influence of the material preparation method on the reflectivity was compared. As shown in Figure 9, when *λ* = 300 to 800 nm, the order of the influence of material preparation methods on the surface reflection performance of SPDT was USRP > RP > CM; when *λ* = 1000 to 1500 nm, the order of the influence of material preparation methods on the surface reflection performance of SPDT was USRP > RP > CM. As shown in Figure 10, when *λ* = 600 nm, the surface optical reflectivity of SPDT prepared by the USRP method increased by 4.875%, 4.401%, 0.490%, and 5.116%, respectively, compared with that prepared by the CM method. When *λ* = 1300 nm, compared with the CM method, the surface optical reflectivity of the SPDT prepared by the USRP method increased by 2.722%, 4.066%, 0.416%, and 1.960%, respectively.

Combined with the analysis of Figure 3, this may be due to the soft texture of the 6061 aluminum alloy, which is prone to deformation during processing. Built-up edges tend to be generated at the tool tip, which leads to an uneven surface under the CM-SPDT process, so its optical reflectivity is also low. The USRP process can change the microstructure of the surface layer, produce grain refinement, improve the surface hardness, reduce the processing deformation and the generation of built-up edges, and make the surface flatter. Therefore, the USRP-SPDT method effectively reduced the surface roughness and improved the optical reflection performance, and the reflectivity increased by 5.116%.

In summary, the USRP-SPDT process proposed in this study uses USRP to modify the surface of the workpiece and then performs SPDT on it. Compared with the conventional manufacturing method of 6061 aluminum alloy mirrors, this method adds a process and increases the processing cost. However, this method has great advantages: residual compressive stress is introduced by USRP processing of the workpiece, so that the grain refinement layer is generated inside the workpiece, which improves the hardness and the machinability of the 6061 aluminum alloy workpiece, thus reducing processing deformation and the generation of built-up edges in the manufacturing process of 6061 aluminum alloy mirrors, and improving the reflectivity. Although this method increases the processing cost in practice, it can achieve higher reflectivity at a lower material cost, which may lead to 6061 aluminum alloy mirrors replacing other materials with higher costs in aerospace manufacturing and other fields. This method also has the advantages of prolonging the service life of the tool, expanding the manufacturing method of the mirrors, and reducing the cost of each workpiece.

### 5.2. Effect of Cutting Parameters on Surface Quality Under USRP-SPDT Process

(1) Surface morphology and roughness

**Figure 11:**
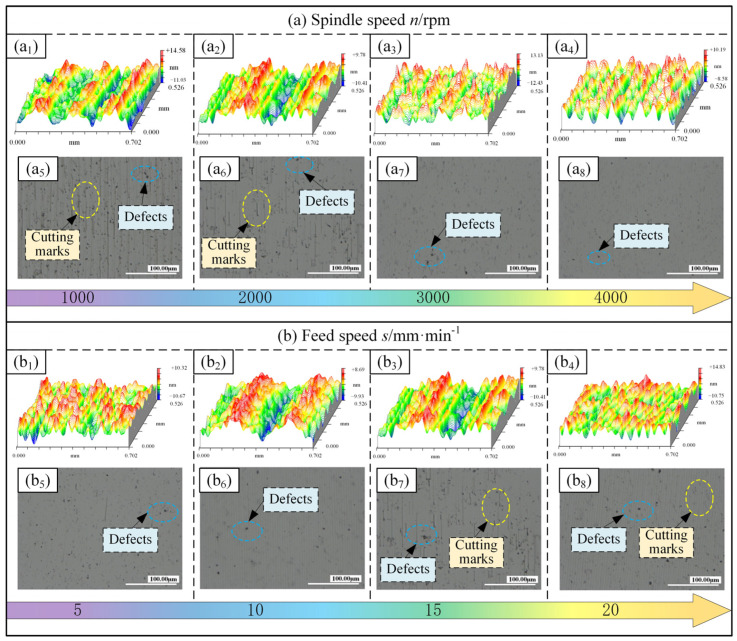
Effect of cutting parameters on surface morphology of USRP-SPDT. (**a_1_**–**a_4_**) are the two-dimensional surface topography diagram at different spindle speeds, and (**a_5_**–**a_8_**) are the three-dimensional surface topography diagram at different spindle speeds. (**b_1_**–**b_4_**) are the two-dimensional surface topography diagram at different feed speeds, and (**b_5_**–**b_8_**) are the three-dimensional surface topography diagram at different feed speeds.

**Figure 12:**
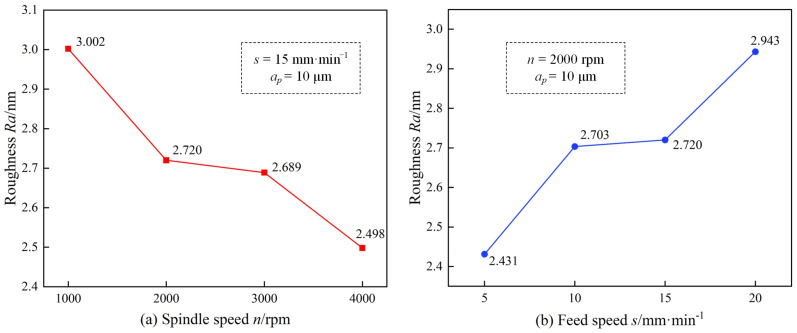
Effect of cutting parameters on surface roughness of USRP-SPDT. *n* refers to the spindle speed, *s* refers to the feed speed, *a_p_* refers to the cutting depth. (**a**) is the influence of spindle speed on surface roughness, (**b**) is the influence of feed speed on surface roughness.

Figure 11a and Figure 12a show the effects of spindle speed on surface morphology and surface roughness, respectively, under the USRP-SPDT method. From Figure 11a, with the increase of rotational speed, the surface morphology became smoother, the frequency of peak–valley alternation increases, and the peak–valley fluctuation gradually decreased. When *n* = 1000 rpm, the image of Figure 11(a_5_) shows that the cutting marks were obvious and there were many processing defects. Combined with Figure 11(a_1_), the frequency of peak–valley alternation was the lowest, and the peak–valley fluctuation was large, reaching 25.61 nm. When *n* = 4000 rpm, the image of Figure 11(a_8_) shows that the cutting marks were hardly observed, and the surface was relatively flat. Combined with Figure 11(a_4_), the frequency of peak–valley alternation was the highest, and the peak–valley fluctuation was small, reaching 18.77 nm. As shown in Figure 12a, the surface roughness decreased with the increase of spindle speed. When *n* = 1000 rpm, the surface roughness was higher (*Ra* = 3.002 nm); when *n* = 4000 rpm, the surface roughness was the lowest (*Ra* = 2.498 nm). The surface roughness at *n* = 4000 rpm was 16.79% lower than at *n* = 1000 rpm. The reason for these results is that in the feed direction, with the increase of the spindle speed, the number of cutting times in the same turning distance increases, the tool tip trajectory is more dense, the overlap area of the tool tip contour increases, and the material removal rate increases, thereby reducing the fluctuation of the peaks and valleys, making the surface morphology gentle, and decreasing the surface roughness.

Figure 11b and Figure 12b show the effects of feed speed on surface morphology and surface roughness, respectively, under the USRP-SPDT method. From Figure 11b, with the increase of feed rate, the surface morphology became rougher, the frequency of alternating peaks and valleys decreased, and the fluctuation of peaks and valleys gradually increased. When *s* = 5 mm/min, the image of Figure 11(b_5_) shows that the cutting marks were not easy to distinguish, and the processing defects were fewer. Combined with Figure 11(b_1_), the frequency of peak–valley alternation was higher, and the peak–valley fluctuation was lower, reaching 20.99 nm. When *s* = 20 mm/min, from the image of Figure 11(b_8_), the cutting marks were evenly distributed, and there were many machining defects. Combined with Figure 11(b_4_), the frequency of peak–valley alternation was low, and the peak–valley fluctuation was large, reaching 25.58 nm. As shown in Figure 12b, the surface roughness increased with the increase of feed speed. When *s* = 5 mm/min, the surface roughness was the lowest (*Ra* = 2.431 nm); when *s* = 20 mm/min, the surface roughness was higher (*Ra* = 2.943 nm). The surface roughness of *s* = 20 mm/min was 21.06% higher than that of *s* = 5 mm/min. The reasons is that in the feed direction, with the increase in feed speed, the cutting time in the same turning distance is shorter, the number of workpiece rotations is reduced, the tool tip trajectory is sparser, the overlap area of the tool tip contour is reduced, and the material removal rate is reduced, which makes the peak–valley fluctuation increase and the surface roughness increase.

(2) Surface optical reflection properties

**Figure 13:**
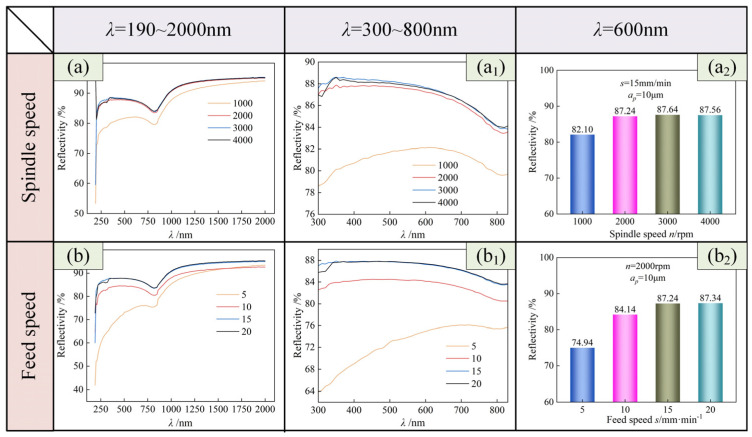
Effect of cutting parameters on the optical reflection performance of USRP-SPDT surfaces. *λ* is the wavelength. (**a**,**b**) are the results of reflectivity detection in the band of 190–2000 nm. (**a_1_**,**b_1_**) are the detection results of reflectivity in the band of 300–800 nm. (**a_2_**,**b_2_**) are the detection results of reflectivity at 600 nm.

Figure 13 shows the measurement results of the surface optical reflection performance under different cutting parameters. The reflection performance was analyzed in the range of *λ* = 300 to 800 nm. A reflectivity value of *λ* = 600 nm was selected as the reference to compare the influence of cutting parameters on reflectivity.

As shown in Figure 13(a_1_), in the range of *λ* = 300 to 800 nm, the surface reflection performance of *n* = 1000 rpm was significantly lower than that of the other three groups, and the reflection performance of the other three groups was not much different. Relatively speaking, the surface reflection performance of *n* = 3000 rpm was better. As shown in Figure 13(a_2_), when *λ* = 600 nm, the surface reflectivity increased first and then stabilized with the increase of spindle speed. When *n* = 1000 rpm, the surface reflectivity was the smallest (82.10%), and when *n* = 3000 rpm, the surface reflectivity was the largest (87.64%). The surface reflectivity of *n* = 3000 rpm was 5.54% higher than that of *n* = 1000 rpm. This shows that increasing the spindle speed can improve the surface reflectivity of the workpiece. This may be because with the increase of spindle speed, within the same turning distance in the feed direction, the amount of material removal increases, making the surface smoother, which helps the light to reflect more evenly, making the surface reflectivity increase. The subsequent surface reflectivity showed a stable trend, which may be due to the microstructure of the surface and other factors, resulting in the absorption and consumption of part of the energy of the light, so the increase of the surface reflectivity is small and tends to be gentle.

As shown in Figure 13(b_1_), in the range of *λ* = 300 to 800 nm, the surface reflection performance of *s* = 5 mm/min was significantly lower than that of the other three groups, and the surface reflection performance of *s* = 20 mm/min was the best. As shown in Figure 13(b_2_), when *λ* = 600 nm, with the increase of feed speed, the surface reflectance showed an increasing trend. When *s* = 5 mm/min, the surface reflectivity was the smallest (74.94%). When *s* = 20 mm/min, the surface reflectivity was the largest (87.34%). The surface reflectivity of *s* = 20 mm/min was 12.40% higher than that of *s* = 5 mm/min. This shows that an increase of feed rate can improve the surface reflectivity of a workpiece. This may be because with the increase of feed speed, in the same turning distance, the number of turning times is reduced, the processing marks are reduced, the frequency of peak–valley fluctuation is reduced, and the light energy of diffuse reflection is reduced, so that the optical reflectivity is increased.

In summary, the surface roughness of the workpiece was inversely proportional to the spindle speed and proportional to the feed speed. The surface reflectivity of the workpiece was proportional to the spindle speed and feed speed.

## 6. Conclusions

In this study, the core idea was to change the microstructure of an aluminum alloy, and the USRP-SPDT processing method was proposed. The feasibility and effectiveness of the method were verified by experiments, which has expanded the research data on reflector mirror processing technologies. The conclusions are as follows:

(1) Compared with CM, USRP can effectively reduce grain size and increase surface hardness. When the frequency was 19 kHz and the amplitude was 4 μm, the grain size decreased by 43.40% and the hardness increased by 10.09%. In terms of the reduction of grain size, the USRP process is superior to the RP process, and the RP process is superior to the CM process.
(2) Compared with SPDT processing, the USRP-SPDT method can effectively reduce surface roughness and improve the surface optical reflection performance. When *n* = 2000 rpm, *s* = 20 mm/min, USRP-SPDT achieved the highest increase in the reflectivity of the visible light band, reaching 5.116%; when *n* = 2000 rpm, *s* = 15 mm/min, USRP-SPDT achieved the highest increase in infrared light band reflectivity, reaching 4.066%.
(3) Under the USRP-SPDT condition, the surface roughness of a workpiece was found to be inversely proportional to the spindle speed and proportional to the feed speed. The surface reflectivity of the workpiece was proportional to the spindle speed and feed speed.

## Figures and Tables

**Table 1:** Aluminum alloy material parameters.

Chemical Composition	Si	Fe	Cu	Mn	Mg	Cr	Zn	Ti	Al
Mass fraction (%)	0.4–0.8	0.7	0.15–0.4	0.15	0.8–1.2	0.04–0.35	0.25	0.15	margin

**Table 2:** Preparative regimen.

Job Number	Rolling Parameters	Frequency/kHz	Amplitude/μm	Working Condition
1~4	No	0	0	CM
5~8	*n* = 2000 rpm, *s* = 60 mm/min, *F* = 600 N	0	0	RP
9~15	*n* = 2000 rpm, *s* = 60 mm/min, *F* = 600 N	19	4	USRP

**Table 3:** Geometrical parameters of the tool.

Shape	Thickness /mm	Accuracy	Nose Radius /mm	Rake Angle /°	Relief Angle /°
Triangle	3.3	G	1.2	0	15

**Table 4:** Material preparation experiment.

Serial Number	Spindle Speed *n*/rpm	Feed Speed *s*/mm·min^−1^	Cutting Depth *a_p_*/μm	Material Preparation Method
1	1000	15	10	CM
2	1000	15	10	RP
3	1000	15	10	USRP
4	2000	15	10	CM
5	2000	15	10	RP
6	2000	15	10	USRP
7	3000	15	10	CM
8	3000	15	10	RP
9	3000	15	10	USRP
10	2000	20	10	CM
11	2000	20	10	RP
12	2000	20	10	USRP

**Table 5:** USRP-SPDT experiment.

Serial Number	Spindle Speed *n*/rpm	Feed Speed *s*/mm·min^−1^	Cutting Depth *a_p_*/μm	Material Preparation Method
1	1000	15	10	USRP
2	2000	15	10	
3	3000	15	10	
4	4000	15	10	
5	2000	5	10	
6	2000	10	10	
7	2000	15	10	
8	2000	20	10	

## Data Availability

The original contributions presented in this study are included in the article. Further inquiries can be directed to the corresponding author.
